# Prothrombinex®‐VF in chronic liver disease: Friend or foe?

**DOI:** 10.1111/1742-6723.14058

**Published:** 2022-08-22

**Authors:** Akmez Latona, Kate Hill, Aurelia Connelly, Katherine Stuart, Peter Wood

**Affiliations:** ^1^ Department of Emergency Medicine Ipswich Hospital Ipswich Queensland Australia; ^2^ Department of Emergency Medicine Princess Alexandra Hospital Brisbane Queensland Australia; ^3^ Department of Emergency Medicine St Vincent's Private Hospital Toowoomba Queensland Australia; ^4^ The University of Queensland Brisbane Queensland Australia; ^5^ Department of Haematology Princess Alexandra Hospital Brisbane Queensland Australia; ^6^ Griffith University Brisbane Queensland Australia; ^7^ Department of Gastroenterology and Hepatology Princess Alexandra Hospital Brisbane Queensland Australia; ^8^ Department of Haematology Calderdale and Huddersfield NHS Foundation Trust Huddersfield England

**Keywords:** accelerated intravascular coagulation and fibrinolysis, acute on chronic liver failure, chronic liver disease, disseminated intravascular coagulopathy, liver cirrhosis, prothrombin complex concentrate, Prothrombinex®‐VF

## Abstract

**Objective:**

Management of coagulopathy in chronic liver disease (CLD) poses a challenge for critical care physicians. Prothrombinex®‐VF is a low‐volume product with rapid onset of action. Evidence for its efficacy and safety in CLD is limited and cases of acute intravascular coagulation and fibrinolysis (AICF) and/or disseminated intravascular coagulation (DIC) have been reported. Our objective was to evaluate the role of Prothrombinex®‐VF in reversal of coagulopathy and the incidence AICF/DIC, thromboembolic events and mortality.

**Methods:**

This was a retrospective, multi‐centre study of Prothrombinex®‐VF use in CLD across 11 hospitals over a 2‐year period, excluding those on therapeutic anticoagulation. Patients were subclassified into acute on chronic liver failure (ACLF), acute decompensation (ADC) and compensated cirrhosis. Reversal of coagulopathy was defined as international normalised ratio (INR) <1.5× upper limit normal (ULN), prothrombin time <1.5× ULN, activated partial thromboplastin time <1.5× ULN and fibrinogen >1 g/L. Markers of AICF/DIC were recorded.

**Results:**

Thirty CLD patients were included, and the median model for end‐stage liver disease score was 23.5. Acute bleeding was the most common indication for Prothrombinex®‐VF (60%). All had baseline coagulopathy and the majority did not achieve reversal. Key indicators of AICF/DIC were mainly observed in those with ACLF; bleeding from mucosa or lines (53%), worsening hypofibrinogenaemia (60%), worsening thrombocytopaenia (60%). The ADC and compensated cirrhosis groups were relatively unaffected. Incidence of venous thromboembolism was 6%. Overall mortality was 43% and 70% in ACLF.

**Conclusion:**

Prothrombinex®‐VF did not lead to meaningful reversal of coagulopathy and should be used with caution in CLD. Patients with ACLF were more likely to develop AICF/DIC following Prothrombinex®‐VF, although the association is uncertain. Further studies are needed to evaluate the safety and efficacy of Prothrombinex®‐VF use in CLD.

## Graphical abstract

Management of coagulopathy in chronic liver disease poses a challenge for critical care physicians. Prothrombinex‐VF® is a low‐volume product with rapid onset of action. Evidence for its efficacy and safety in chronic liver disease is limited and cases of acute intravascular coagulation and fibrinolysis and/or disseminated intravascular coagulation have been reported. We evaluate the role of Prothrombinex‐VF® in reversal of coagulopathy and the incidence acute intravascular coagulation and fibrinolysis/disseminated intravascular coagulation, thromboembolic events and mortality.

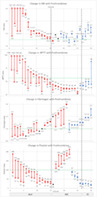

**Key findings**

- INR does not predict bleeding risk in chronic liver disease as haemostasis is rebalanced.
- In advanced disease, there is often accelerated intravascular coagulation and fibrinolysis, similar to DIC.
- Prothrombinex®‐VF is contraindicated in DIC.
- Prothrombinex®‐VF does not lead to meaningful reversal of coagulopathy in chronic liver disease.
- In acute on chronic liver failure, progression to a state of severe DIC following Prothrombinex®‐VF use is increased, although the association is uncertain.

## Introduction

Chronic liver disease (CLD) is a major burden on the healthcare system in Australia^1^ and management of haemostasis frequently poses a challenge for critical care physicians. Coagulopathy in cirrhosis is complex and exists as precariously ‘rebalanced’ haemostasis that can shift to either a prothrombotic or bleeding state in different clinical situations. Table 1 summarises the key changes.^1^, ^2^ Expert guidance exists for the management of coagulopathy in CLD; however, there is a lack of evidenced‐based guidelines.^2^, ^3^ The role of prothrombin concentrates, such as Prothrombinex®‐VF, is uncertain in this population.^3^

**TABLE 1:** Summary of haemostatic changes in chronic liver disease

Haemostasis	Chronic liver disease	
Response to injury	Increases clot promotion	Increases bleeding risk
Primary	Elevated vWF, reduced ADAMTS‐13	Thrombocytopaenia
Vasoconstriction	Platelet activation	Platelet dysfunction
Platelet adhesion	Endothelial activation	Anaemia
Platelet aggregation		
Secondary Activation of coagulation cascade (thrombin burst)	Low anticoagulant factors (↓Protein C, S, antithrombin) ↑FVIII	Low procoagulant factors (↓FII, V, VII, IX, X, XI, XIII) ↓Fibrinogen
Formation of fibrin	Fibrin clot permeability	↓Rate of fibrin polymerisation
Tertiary	Plasminogen	↑tPA
Activation of fibrinolysis	↓PAI	↓Alpha2‐antiplasmin
Lysis of the clot		↓TAFI

ADAMTS‐13, a disintegrin and metalloproteinase thrombospondin type 1 motif; F, factor; PAI‐1, plasminogen activator inhibitor 1; TAFI, thrombin activatable fibrinolysis inhibitor; tPA, tissue plasminogen activator; vWF, von Willebrand factor.

Acute on chronic liver failure (ACLF) is a relatively new defined entity, associated with organ failure and high mortality.^4^ In ACLF, coagulation frequently tends towards a bleeding phenotype with onset of a systemic inflammatory response and endothelial activation, often worsened by sepsis.^5^ The increased formation of poor‐quality clot and fibrinolysis leads to a condition that resembles disseminated intravascular coagulation (DIC), labelled ‘accelerated intravascular coagulation and fibrinolysis’ (AICF). AICF is observed in 30% of patients with moderate to severe liver failure^4^ and correlates with mortality and severity of liver disease. Clinical manifestations include bleeding from mucosae and intravascular lines accompanied by laboratory findings of elevated D‐dimer level, low fibrinogen, elevated prothrombin time (PT)/activated partial thromboplastin time (APTT) and progressive thrombocytopaenia, all of which are observed in DIC. Predictors of bleeding in ACLF, include fibrinogen level <0.6 g/L, platelet count <30 × 10^9^/L and APTT >100 s. In the acute setting, drawing a distinction between AICF and DIC is difficult.

In CLD, conventional laboratory coagulation tests do not reflect the derangement in haemostasis, or accurately predict the risk of bleeding.^6^ The greatest misconception is the use of international normalised ratio (INR) in the clinical assessment of bleeding risk in patients with liver disease. INR increases modestly with synthetic dysfunction; however, it does not reflect bleeding risk because thrombin generation is preserved in compensated cirrhosis. Indeed, INR has only been validated for warfarinised patients, although unvalidated attempts have been made to develop an INR_(LIVER)._ ^7^ Hypofibrinogenaemia and degree of thrombocytopaenia are better indicators of bleeding risk during episodes of hepatic decompensation.^8^ Global coagulation assays such as rotational thromboelastometry and thromboelastography are potentially more accurate in reflecting the clinical bleeding risk and guiding targeted replacement therapy by assessing the interactions between procoagulant and anticoagulant factors, platelets and the fibrinolytic system. However, these assays have not yet been validated for routine use in CLD and prospective studies are currently underway evaluating their role in clinical practice.^9^

Management of coagulopathy in the acute bleeding and peri‐procedural environments is challenging, with no well‐defined reversal targets. Established clinical guidelines recommend against the reversal of INR to <1.5 for procedures and the evidence to support this target is lacking in the acute bleeding patient.^2^, ^10^, ^11^ Correction of coagulopathy is a crucial component of resuscitation of the bleeding patient while achieving source control. Traditional blood products such as cryoprecipitate and fresh frozen plasma (FFP) are often used in the acute bleeding situation, and less frequently vitamin K (with its delayed effect) and tranexamic acid. Large volumes of FFP are often required to correct factor deficiencies and result in increased portal pressures potentially further increasing the risk of gastrointestinal bleeding.^12^

Prothrombinex®‐VF is an Australian produced four‐factor prothrombin complex concentrate (4F‐PCC) which contains factors II, IX, X and low levels of factor VII, with final overall clotting factor concentration 25 times high than normal plasma and marked prothrombotic activity through generation of the thrombin burst.^13^ It is indicated for reversal of vitamin K antagonists, anti‐Xa inhibitors (rivaroxaban and apixaban) and is contraindicated in DIC and active thrombosis.^14^ As most gastrointestinal bleeding in cirrhosis is driven by portal hypertension, the use of Prothrombinex®‐VF is appealing because smaller volumes are administered compared to FFP.^15^ However, in advanced cirrhosis, DIC can go unrecognised as a contraindication to the use of the product.^16^

We performed a retrospective cohort study of the use of Prothrombinex®‐VF in CLD patients across Metro South and West Moreton Health Services. The aim of the present study was to assess the effect of Prothrombinex®‐VF on clinical and laboratory markers of haemostasis in CLD patients and measure the secondary outcomes of episodes of thrombosis, AICF/DIC and mortality.

## Methods

### Data collection

The present study was approved by the Metro South and West Moreton Health Research Ethics Committees. Data were obtained from patients' electronic medical records and laboratory information systems. Patient demographic, clinical, laboratory parameters and use of haemostatic agents were recorded (Appendix S1).

### Study design

This was a multi‐centre, retrospective, observational study of all adult patients who received Prothrombinex®‐VF at Metro South and West Moreton Hospitals and Health Services, Queensland, Australia, between January 2018 and December 2020 with the goal of evaluating its use specifically in patients with CLD.

Inclusion criteria is CLD of any aetiology.

Exclusion criteria are as follows:

- Congenital or acquired coagulation factor deficiency;
- Patients on anticoagulants (vitamin K antagonists and direct oral anticoagulants); use of antiplatelet agents was not an exclusion;
- Peri‐operative liver transplant;
- Pre‐procedural reversal of coagulopathy not related to underlying CLD;
- Non‐liver related coagulopathy (sepsis, trauma, multi‐organ failure, pregnancy, toxicology, coagulopathy of unclear cause).

Patients with CLD were sub‐classified into ACLF, acute decompensated cirrhosis (ADC) and compensated cirrhosis (CC). ACLF was defined according to the EASL criteria.^4^ Model for end‐stage liver disease (MELD), Child‐Pugh score and Chronic Liver Failure Consortium Acute on Chronic Liver Failure (CLIF‐C ACLF) scores were calculated for each patient.

#### Definitions

Acute on chronic liver failure:

- Presence of at least one organ failure in patients with ADC; creatinine >177 μmol/L for renal failure, total bilirubin level >205 μmol/L for liver failure, INR >2.5 for coagulation failure, West‐Haven hepatic encephalopathy grade 3–4 for brain failure, use of vasopressors for circulatory failure and PaO_2_/FiO_2_ <200 or need for mechanical ventilation for respiratory failure.^4^

Acute decompensated cirrhosis:

- ADC refers to the development of ascites, encephalopathy, gastrointestinal haemorrhage, or any combination of these features in patients with cirrhosis.^17^

Compensated cirrhosis:

- CC refers to the absence of the above clinical features of decompensation in patients with cirrhosis.^17^

### Study outcomes

#### Primary endpoint

The primary outcome was reversal of coagulation markers within 72 h after administration of Prothrombinex®‐VF, as defined by:

- INR <1.5,
- PT <20 s,
- APTT <59 s (1.5× upper limit normal) and
- Fibrinogen >1 g/L.

#### Secondary endpoints

1. Evidence of AICF and/or DIC, as defined by:
  - Elevated D‐dimer,
  - Worsening of each of the following parameters by ≥15%:
    - Hypofibrinogenaemia if fibrinogen <1 g/L,
    - Thrombocytopaenia if platelet <100 × 10^9^/L,
    - Prolonged PT >20 s and
    - APTT of >59 s.
  - Fibrinogen discordance >0.3 (defined as derived minus clottable fibrinogen) and
  - Red cell fragmentation.
2. Documented venous or arterial thromboembolic event within 30 days, as recorded in patient medical records or radiology.
3. Overall survival.

### Analysis

Data were analysed in Microsoft Excel and presented as descriptive statistics and scatter plots. Sample size was insufficient to perform inferential statistical analysis.

## Results

### Patient characteristics

**Figure 1:**
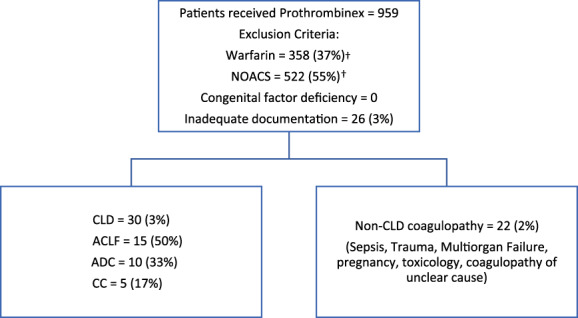
Cohort diagram. †Four patients on warfarin and one on direct oral anticoagulants with underlying chronic liver disease (CLD) were excluded from the analysis. ACLF, acute on chronic liver failure; ADC, acute decompensated cirrhosis; CC, compensated cirrhosis.

Prothrombinex®‐VF was administered to 959 adults during the 2‐year study period and 30 (3%) patients had CLD (Fig. 1). Patient demographics, liver disease aetiology and severity, type of bleeding and hospital location of administration of Prothrombinex®‐VF are presented in Table 2.

**TABLE 2:** Demographics and clinical characteristics of patients with chronic liver disease (CLD)

	Number of patients, *n* (%)
Total number of patients	30
Sex, *n* (%)	
Male	21 (70)
Female	9 (30)
Age (years), median	58.5
Aetiology of CLD, *n* (%)	
Alcohol	17 (56)
Chronic viral hepatitis B or C	7 (24)
Other	6 (20)
Cirrhosis subgroup, *n* (%)	
ACLF	15 (50)
ADC	11 (37)
CC	4 (13)
Liver disease severity, median (range)	
Child‐Pugh	10 (7–13)
MELD	23 (16–40)
CLIF‐C ACLF	53 (43–81)
Indication for Prothrombinex®‐VF, *n* (%)	
Gastrointestinal bleeding	11 (37)
Vascular bleeding	3 (10)
Soft tissue bleeding	3 (10)
Multi‐trauma	1 (3)
Pre‐procedure INR reversal	8 (27)
Severe coagulopathy without bleeding	3 (10)
Inadequate documentation	1 (3)
Hospital location of administration, *n* (%)	
ED	9 (30)
ICU	13 (43)
Medical ward	5 (17)
Surgical ward	3 (10)

ACLF, acute on chronic liver failure; ADC, acute decompensated cirrhosis; CC, compensated cirrhosis; CLIF‐C ACLF, Chronic Liver Failure Consortium Acute on Chronic Liver Failure; MELD, model for end‐stage liver disease.

The most common aetiology of CLD was alcohol‐related liver disease (53%) with many patients having more than one cause. The median Child‐Pugh score was 10 (range 7–13) and MELD of 23 (range 16–40). ACLF criteria was met in 50% of patients with a median CLIF‐C score of 53 (range 43–81). No patient was on dual antiplatelet therapy and only 10% of patients were on aspirin. Management of acute bleeding was the indication for Prothrombinex®‐VF use in 57% with the majority presenting with gastrointestinal bleeding (37%) and it was administered predominantly in the ICU (43%) and ED (30%). In 37% of patients, Prothrombinex®‐VF was used to reverse INR pre‐procedural or in the absence of clinical bleeding.

### Use of Prothrombinex®‐VF, haemostatic agents and blood products

Table 3 summarises the haemostatic interventions and source control of bleeding in the entire cohort and subgroups. The median dose of Prothrombinex®‐VF was 25 IU/kg in the whole cohort. No patient received fibrinogen concentrate, factor VIIa or desmopressin. Although all products were prescribed simultaneously, there was often a delay in administration of cryoprecipitate and FFP until after Prothrombinex®‐VF. Vitamin K and tranexamic acid were used infrequently. No major differences were observed in the use of blood products between the three cirrhosis sub‐groups. Control of bleeding was successful in 16 (53%) patients: 12 patients requiring endoscopic therapy, two had surgery, one radiological embolisation and a single patient had endometrial ablation.

**TABLE 3:** Use of Prothrombinex®‐VF, haemostatic agents and blood products

Haemostatic intervention	Total cohort (*n* = 30)	ACLF (*n* = 15)	ADC (*n* = 11)	CC (*n* = 4)
Prothrombinex®‐VF (IU/kg), median (range)	25 (6–63)	30 (8–51)	24 (6–53)	42 (30–63)
Vitamin K, *n* (%)	16 (53)	6	7	3
Tranexamic acid, *n* (%)	3 (10)	1	0	2
Source control of bleeding, *n* (%)	16 (53)	7	8	1
Blood products (U), *n* (%)				
Cryoprecipitate	20 (67)	11 (73)	6 (55)	3 (75)
Pooled platelet	16 (53)	10 (67)	3 (27)	4 (75)
PRBC	24 (80)	12 (80)	9 (82)	5 (75)
FFP	14 (47)	9 (60)	4 (26)	1 (25)

ACLF, acute on chronic liver failure; ADC, acute decompensated cirrhosis; CC, compensated cirrhosis; FFP, fresh frozen plasma; PRBC, packed red blood cells.

### Primary endpoint

**Figure 2:**
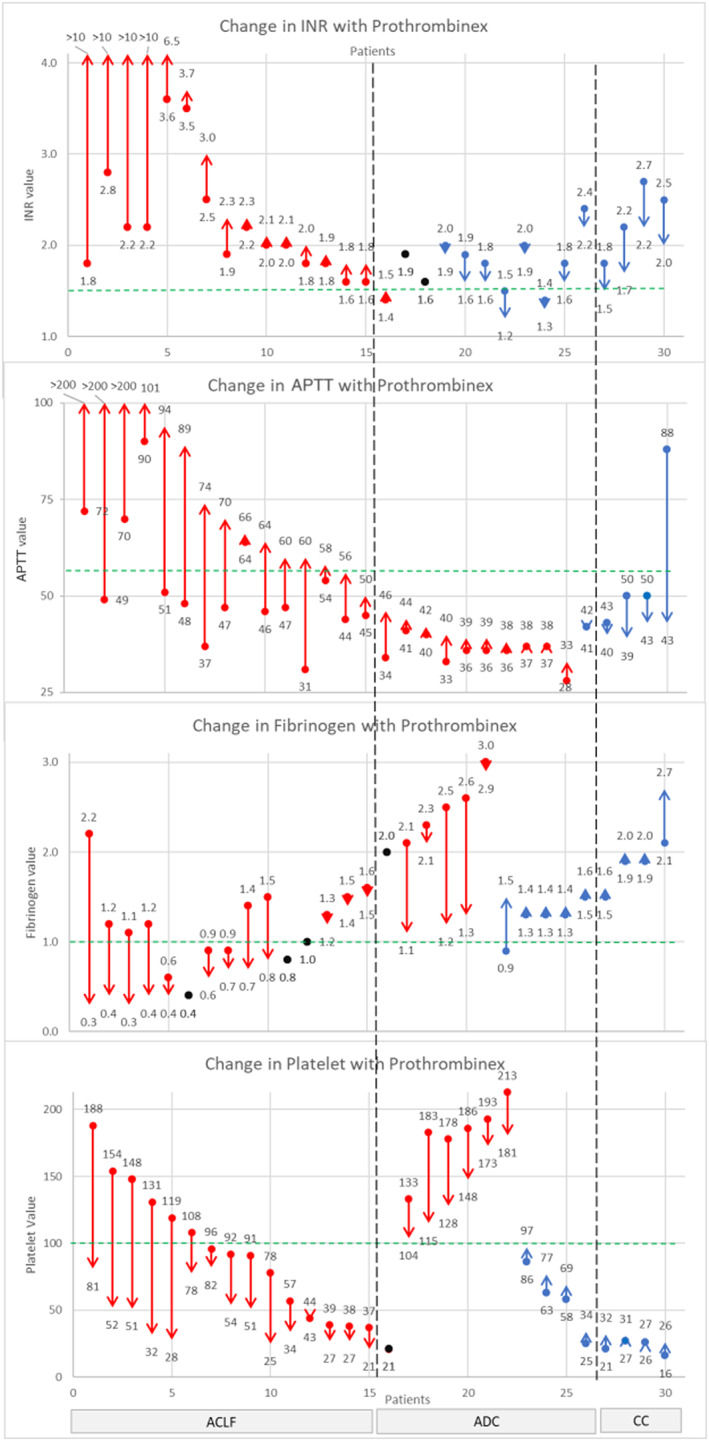
Demonstrates individual patient trends in international normalised ratio (INR), activated partial thromboplastin time (APTT), fibrinogen and platelet count within 72 h of Prothrombinex®‐VF administration. The vertical red lines represent worsening of the coagulation marker and the vertical blue lines represent improvement of the coagulation marker. The horizontal green dotted lines represent the threshold for reversal of coagulation marker. Patients 1–15 had acute on chronic liver failure (ACLF), patients 16–26 had acute decompensated cirrhosis (ADC) and patients 27–30 had compensated cirrhosis (CC).

Most patients had disturbed markers of coagulation at baseline and failed to achieve reversal of coagulopathy following Prothrombinex®‐VF administration (Fig. 2). In five patients from ACLF subgroup, there is marked prolongation of PT, APTT and severe hypofibrinogenaemia. In the remaining majority of patients, their coagulation markers did not change significantly following Prothrombinex®‐VF.

### Secondary endpoints

#### AICF/DIC

Nine (30%) of the entire CLD and 53% of the ACLF sub‐group of patients had clinical evidence of AICF with bleeding from mucosae and intravascular lines, with laboratory evidence of worsening coagulopathy (Table 4). Only six patients, all with ACLF had a D‐dimer test. Worsening thrombocytopaenia was seen in all cirrhosis sub‐groups.

**TABLE 4:** Markers of AICF/DIC following Prothrombinex®‐VF administration

Evidence of AICF/DIC	CLD, *n* = 30, *n* (%)	ACLF, *n* = 15, *n* (%)	ADC, *n* = 11, *n* (%)	CC, *n* = 4, *n* (%)
Bleeding from mucosae/intravascular lines	9 (30)	8 (53)	0	0
Elevated D‐dimer	6 (20)	6 (40)	0	0
Worsening PT >20 s†	7 (23)	7 (15)	0	0
Worsening APTT >59 s†	10 (33)	10 (15)	0	0
Worsening thrombocytopaenia <100 × 10^9^/L†	14 (47)	9 (60)	4 (36)	1 (25)
Worsening hypofibrinogenaemia <1 g/L†	10 (33)	9 (60)	1 (9)	0
Fibrinogen discordance ≥0.3‡	10 (33)	9 (60)	1 (9)	0

† ≥15% change of the above coagulation markers.

‡ Fibrinogen discordance = derived fibrinogen (fib d) − clottable fibrinogen (fib c).

ACLF, acute on chronic liver failure; ADC, acute decompensated cirrhosis; AICF, acute intravascular coagulation and fibrinolysis; APTT, activated partial thromboplastin time; CC, compensated cirrhosis; CLD, chronic liver disease; DIC, disseminated intravascular coagulation; PT, prothrombin time.

#### Thromboembolic events

Two patients were diagnosed with venous thromboembolism, both lower limb deep vein thrombosis, within 5 days after Prothrombinex®‐VF administration. An additional two patients were diagnosed with thrombo‐emboli prior to administration of Prothrombinex®‐VF, including one patient with portal vein thrombosis and a second with an arterial lower limb embolus resulting in ischaemia. There were no cardiovascular or cerebrovascular adverse events within 30 days of Prothrombinex®‐VF administration.

#### Mortality

All‐cause‐in‐hospital mortality was 43%. Mortality rates were highest in ACLF (60%), with six of nine deaths related to multiorgan failure, the remainder were uncontrolled haemorrhage, sepsis and end‐stage liver disease. In ADC, the mortality rate was 18% with two deaths related to uncontrolled haemorrhage and sepsis. There were low numbers in the CC group; two patients died from multiorgan failure following hypovolaemic shock and sepsis.

## Discussion

There was a small (3%) incidence of off‐label use of Prothrombinex®‐VF for treatment of cirrhotic coagulopathy across Metro South and West Moreton Health Services and it was mainly used by emergency and intensive care physicians in the management of bleeding patients. We could not demonstrate benefit for use in patients with CLD; in most cases, there was minimal impact on correcting the coagulation profile. A significant proportion of patients with ACLF (53%) developed a DIC‐like picture which in many cases was unsalvageable with haemostatic measures. The incidence of thrombosis (6%) was not increased above the predicted incidence observed in CLD and similar to that reported by others.^18^, ^19^ Our patient cohort had advanced liver disease with median MELD and CLIF‐C ACLF scores of 23 and 53, respectively, and hence, not surprisingly we observed a high all‐cause‐in‐hospital mortality of 43%.

Our study is unique in that we have subdivided our CLD cohort of patients into distinct groups of ACLF, ADC and CC. This separation demonstrated that patients with CC or ADC given Prothrombinex®‐VF do not appear to be at the same risk of AICF compared to patients with ACLF. However, we propose that perhaps there is no role for the use of Prothrombinex®‐VF in CC and ADC patients, as it did not reverse coagulopathy in these sub‐groups. Up to 60% of patients with ACLF had multiple markers of DIC following Prothrombinex®‐VF administration, manifest by fibrinogen discordance (a surrogate measure for elevated fibrin degradation products), worsening PT, hypofibrinogenaemia and worsening thrombocytopaenia. Progressive thrombocytopaenia was present in all cirrhosis subgroups, but was most frequently observed in ACLF (60%), reflecting the multifactorial nature of this marker which, for many reasons can worsen acutely, one of which is DIC.^8^ Five patients had a profound coagulopathy raising the suspicion that these patients may have had unrecognised DIC prior to receiving Prothrombinex®‐VF. Other possible contributing factors include systemic inflammatory response, consumptive coagulopathy related to massive blood loss and worsening liver function as part of multi‐organ failure.^8^

The present study highlighted the frequent disconnect between the laboratory and the treating clinician. In 10 (33%) patients, there was discordance between derived fibrinogen (an estimate mathematically derived from prothrombin time kinetics) and clottable fibrinogen (the functional assay). When the difference between these two values is ≥0.3, this is because of the presence of fibrin degradation products. Although some Queensland Pathology laboratories will automatically test for D‐dimer in this situation, this is not mandated or universal across all hospitals.^20^

Dosing of Prothrombinex®‐VF for vitamin‐K antagonist reversal is based on evidence using current and target INR levels and patient weight.^14^ The wide variation in dosing strategy in our cohort reflects the lack of evidence to guide its use in CLD. In many cases, although blood products were prescribed simultaneously with Prothrombinex®‐VF, the latter was available almost immediately. The delay in administering cryoprecipitate because of thawing meant that in most cases, Prothrombinex®‐VF was administered prior to correction of hypofibrinogenaemia. Correction of hypofibrinogenaemia and optimisation of coagulation using other haemostatic products prior to Prothrombinex®‐VF in CLD may improve the safety of its use. There is clear evidence to support achieving a fibrinogen level of >1 g/L to enable sufficient clot formation^21^ and this is supported by the success of global assays of haemostasis which have clear targets for early correction of hypofibrinogenaemia. It is important to target all potential reversible deficiencies; vitamin K deficiency is common in patients with CLD and contributes to prolongation of PT and APTT. Vitamin K was administered in 53% of the total cohort, and although its action peaks at 24–48 h, it may have an additional role. Very few patients in the present study were given tranexamic acid (10%), possibly because of concerns over thrombotic risks in the context of advanced liver disease.

There are no prospective or randomised studies evaluating the role of PCCs in liver disease. There are two retrospective studies evaluating the use of 4F‐PCCs in patients with liver disease. Huang *et al*. suggested that 4F‐PCC to correct coagulopathy was less beneficial in patients with liver disease compared to those without liver disease.^18^ In contrast, Drebes *et al*. evaluated 81 patients with CLD reporting statistically significant reductions in INR before and after 4F‐PCC. However, the median INR level achieved was 1.8 which arguably is still abnormal and of unclear clinical significance because of issues discussed above in its relation to liver disease.^19^ Neither study mentions episodes of DIC or AICF whereas several case studies have reported this phenomena.^15^, ^22^

The present study has several limitations including the retrospective nature and small number of patients, limiting the ability to undertake any robust statistical analysis. Patient numbers were too small to form any firm conclusions between liver disease severity and coagulation markers to predict which patients may be more likely to be at risk of this rare acute DIC‐like coagulopathy. Multi‐organ failure and sepsis are well‐recognised causes of coagulopathy, and frequently seen in ICU;^8^ however, it is rare to see the degree of significant acute coagulopathy observed in the ACLF cohort, suggesting that these patients are a distinct cohort with markedly abnormal coagulation. Furthermore, diagnosing DIC in CLD is difficult and some of the patients in this cohort may have had undiagnosed DIC prior to administration of Prothrombinex®‐VF, which then resulted in worsening of their coagulation profile.^23^

## Conclusion

Prothrombinex®‐VF is contraindicated in DIC, and it should be used with caution in CLD. The present study failed to show a meaningful improvement in coagulopathy with Prothrombinex®‐VF in patients with CLD. In ACLF, a significant proportion of patients progressed to a state of AICF/DIC following Prothrombinex®‐VF administration, although the association is uncertain. Further studies are needed to evaluate the safety and efficacy of Prothrombinex®‐VF use in CLD.

## Supporting information


**Appendix S1.** Supporting information.

## Data Availability

The data that support the findings of this study are available from the corresponding author upon reasonable request.
