# Supplementary material for: Prothrombinex®‐VF in chronic liver disease: Friend or foe?
Source: Emerg Med Australas. 2022 Aug 22;35(1):89–96. doi: 10.1111/1742-6723.14058 (PMC10087488; doi:10.1111/1742-6723.14058)

# *APPENDIX*

Patient medical files were reviewed and the following data were recorded: patient demographics, liver disease aetiology and severity, presence of organ failures, location of use of Prothrombinex®-VF (emergency department, intensive care, operating theatre, medical or surgical ward) and dose in IU/kg, nature of bleeding event, 30 day mortality from all causes, use of anti-platelet drugs, and laboratory coagulopathy indices at 0, 24, 48 and 72 hours after Prothrombinex®-VF administration (D-dimer, prothrombin time (PT), activated partial thromboplastin time (APTT), platelet count, and fibrinogen level). Data on the concurrent use of blood products including fresh frozen plasma (FFP), packed red blood cells (PRBCs), platelets and cryoprecipitate received within 72 hours of Prothrombinex®-VF administration was collected. Other treatments affecting haemostasis including administration of vitamin K, recombinant factor VIIa, tranexamic acid, fibrinogen concentrate, or desmopressin received within 72 hours were recorded.

Change in PT with Prothrombinex®-VF graph:


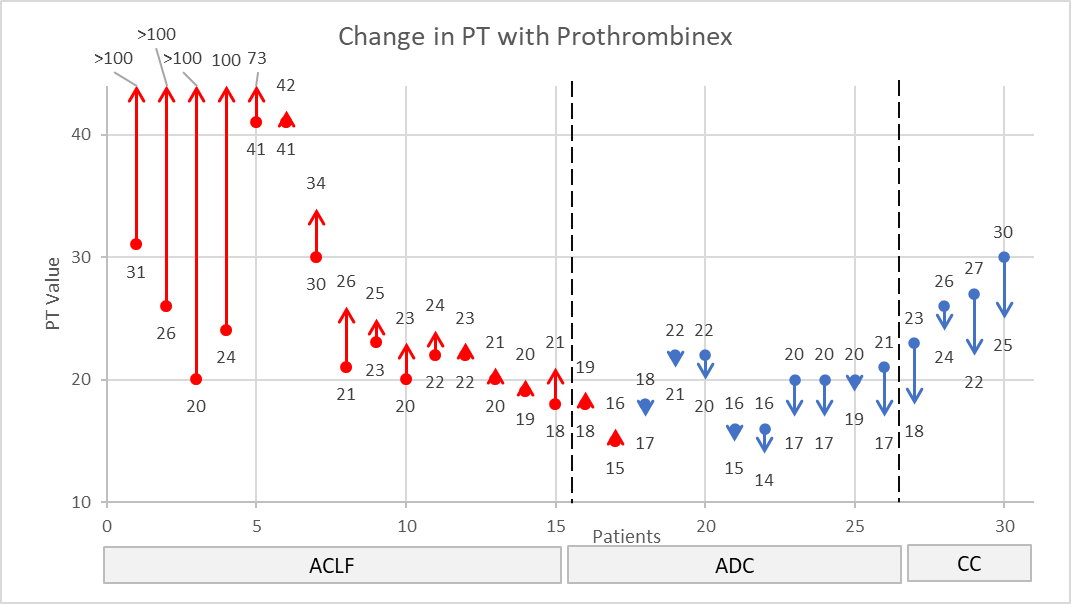

Supplement: Supplementary file 1 — Appendix S1. Supporting information. [file EMM-35-89-s001.docx]
